# ParaRef: a decontaminated reference database for parasite detection in ancient and modern metagenomic datasets

**DOI:** 10.1186/s13059-025-03818-w

**Published:** 2025-10-23

**Authors:** Jonas Niemann, Yuejiao Huang, Liam T. Lanigan, Arve L. Willingham Grijalba, Robert R. Dunn, Martin Sikora, Hannes Schroeder

**Affiliations:** 1 Globe Institute, Faculty of Health and Medical Sciences, University of Copenhagen, Copenhagen, 1350 Denmark; 2 School of Archaeology, Faculty of Humanities, University of Copenhagen, Copenhagen, 2300 Denmark; 3 Microbial Paleogenomics Unit, Institut Pasteur, Université Paris Cité, CNRS UMR 2000, Paris, 75015 France; 4 North Carolina State University, Raleigh, NC 27695-7617 USA

## Abstract

Shotgun metagenomics holds great potential for identifying parasite DNA in biological samples, but its effectiveness is limited by widespread contamination in publicly available reference genomes, which hinders accurate detection. In this study, we systematically quantify and remove contamination from 831 published endoparasite genomes to create ParaRef, a curated reference database for species-level parasite detection. We show that decontamination significantly reduces false detection rates and improves overall detection accuracy. Our study highlights the pervasive issue of contamination in public databases and offers a resource that will enhance the reliability of parasite detection using metagenomics.

**Supplementary Information:**

The online version contains supplementary material available at 10.1186/s13059-025-03818-w.

## Background

Human parasites constitute a major health burden, with common parasitic diseases being a leading cause of death worldwide [1, 2]. For example, malaria, the parasitic disease with the highest estimated annual death toll, causes more than 600,000 deaths annually, according to WHO estimates [3]. Today, parasitic infections primarily affect rural communities in developing countries with limited sanitation and poor water quality. In the past, however, they were likely far more widespread. In Europe, for example, parasitic diseases remained common until the mid-20th century, causing significant morbidity and mortality [4]. The earliest archaeologically attested human parasitic infections date to ~ 30,000 years ago [5], but the recovery of ancient DNA from much older contexts [6] suggests that this can potentially be pushed further back in time. Parasites recovered from archaeological sites can also provide important insights into the health and lifestyles of past human populations [7, 8]. For example, human gastrointestinal parasites, such as the roundworms *Ascaris *spp*.* or *Trichuris *spp*.*, are good indicators of living conditions and general hygiene, while *Taenia *spp*.* or *Opisthorchis *spp*.*, which infect humans through the ingestion of raw or undercooked meat and fish, can inform our understanding of past diets and cooking practices (e.g., [9]). Understanding parasite life cycles and transmission routes is also essential, as it can reveal how closely humans interacted with animals, offering insights into agricultural practices and domestication processes [8, 9].

Traditional methods for detecting parasitic infections include microscopic examination of stool or blood samples, endoscopy, imaging techniques, and molecular assays such as PCR [10–12]. However, these approaches are often limited by the inability to reliably identify parasites at the species level and by the morphological similarities between taxa. Recently, shotgun sequencing has emerged as a powerful alternative for detecting parasites in clinical, ecological, and archaeological settings [13–15]. This approach identifies DNA sequences by comparing them against databases of reference genomes. In archaeological contexts, shotgun sequencing has the added benefit of distinguishing ancient DNA from modern contamination based on characteristic damage patterns, potentially providing insights into the evolutionary history of parasite species. However, this approach relies heavily on the integrity of reference genome databases, which are known to have several limitations, including taxonomic underrepresentation, incorrect taxonomic labelling, and poor-quality reference sequences [16]. Another well-known issue is reference genome contamination [17, 18], which can lead to false-positive identifications.

**Fig. 1:**
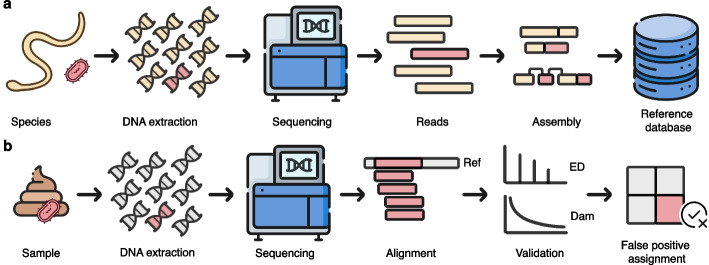
How does reference contamination occur and how does it lead to false positives? **a** Contamination in publicly available reference genomes arises as contaminant DNA (e.g., from the host or the environment) is integrated into reference assemblies. **b** False positive assignments occur as contaminant DNA sequences (e.g., from bacteria) are misaligned to the contaminant sequences in the reference genome. These sequences will show the expected declining edit distance distribution (ED) and, if they are ancient, they will also display the expected ancient DNA damage patterns (Dam), passing validation checks

Reference genome contamination occurs when DNA from other organisms is inadvertently incorporated during genome assembly (Fig. 1). This can lead to false-positive detections, faulty conclusions about horizontal gene transfer, and misdiagnoses in clinical settings. Eukaryotic genomes are especially prone to contamination; for example, Astashyn et al. [19] found that 44% of eukaryotic genomes in GenBank and RefSeq contain contaminant sequences, compared to just 5% of prokaryotic genomes. Contamination often originates from organisms biologically associated with the target (e.g., through diet or the host microbiome) or is introduced during sample processing via reagents, handling, or packaging [19]. Parasite genomes are particularly vulnerable, as parasite samples frequently contain host DNA—and conversely, parasite DNA is sometimes present in host genome assemblies [20, 21]. Notable examples include fish DNA in the salmon louse genome and *Sarcocystis* DNA in the genomes of the thicket tinamou and sperm whale [19]. Such cross-contamination increases the risk of false-positive hits in metagenomic screening. However, despite growing awareness of this issue, there is currently no consensus on how to prevent contamination-related misalignments or evaluate their impact on metagenomic studies.

Several tools have been developed to identify and remove contaminant sequences in newly sequenced genomes [19, 22–24]. For example, Conterminator [23] employs an all-against-all sequence comparison to identify contaminants across taxonomic kingdoms, with a focus on incorrectly labelled sequences in databases like RefSeq and GenBank. By breaking sequences into segments and aligning them, it can detect foreign sequences, even in scaffolds, where contamination may be embedded within contigs. This tool has identified millions of base pairs of contamination across public databases, highlighting the extent of the problem [23]. Another tool, FCS-GX [19], developed as part of NCBI’s Foreign Contamination Screen (FCS) suite of tools, was optimised for speed and efficiency. It can screen genomes in a matter of minutes, identifying contamination with high sensitivity and specificity. FCS-GX has been used to process 1.6 million GenBank assemblies, reducing the contamination in RefSeq genomes to just 0.01% of total bases [19]. However, despite these advances, contamination remains a pervasive issue in many reference genomes—particularly among less well-curated groups such as parasites.

In this study, we screened 831 published endoparasite genomes with FCS-GX [19] and Conterminator [23] to quantify the prevalence and impact of reference sequence contamination. We then compiled a curated, decontaminated parasite genome reference database, ParaRef, and evaluated how it improved parasite detection rates using both simulated and real-world metagenomic datasets. By addressing contamination at the reference level, we report substantial reductions in false-positive detections—without sacrificing true-positive sensitivity—thereby providing a more reliable framework for metagenomic parasite screening in ecological, clinical, and archaeological settings.

## Results

### Contamination in published parasite reference genomes

**Fig. 2:**
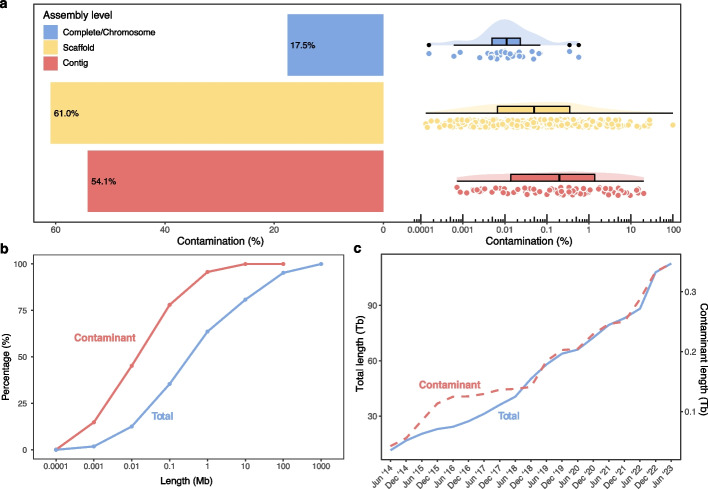
Extent of contamination in published parasite genomes detected with FCS-GX. **a** Proportion of published parasite genomes flagged as contaminated by assembly level (left); Fraction of contaminant bases in each parasite genome (right); **b** Cumulative proportions of contaminant and total number of bases in published parasite genomes plotted by contig length; **c** Cumulative length of contaminant parasite sequences by submission date. The results for Conterminator [23] are shown in Additional File 2: Fig. S1

Out of all 831 parasite genomes included in this study (Additional File 1: Table S1), FCS-GX [19] identified 346,990,249 contaminant bases across 430 genomes, while Conterminator [23] detected 365,285,331 contaminant bases across 801 genomes. Combining the results of both methods, a total of 528,479,404 bases were flagged as contamination in 818 genomes (Additional File 1: Table S2). Over half of contig- or scaffold-level assemblies were found to be contaminated, and in 64 cases the contaminated fraction exceeded 1% of the genome (Fig. 2a). In the most extreme case, the genome of *Elaeophora elaphi*, a nematode infecting red deer, consisted entirely of sequences of the bacterium *Brucella anthropium* (Additional File 1: Table S3). Interestingly, while Conterminator [23] flagged contamination in nearly twice as many genomes as FCS-GX [19], the total number of contaminant bases was comparable between the two methods (Fig. 2; Additional File 1: Table S2; Additional File 2: Fig. S2).

In general, the better the quality of the assembly, the lower the level of contamination (Fig. 2a). Only 17% of complete genomes or genomes assembled to chromosome level were found to be contaminated, with a maximum of just 0.5% of the most contaminated genome deriving from a contaminant. In contrast, over 50% of genomes at scaffold and contig level were contaminated, with 18 genomes containing 10% of contamination or more. Concerning contig length, we found that shorter contigs were more contaminated than longer contigs (Fig. 2b). More than 75% of all detected contamination was detected in contigs shorter than 100 kb, even though such contigs make up just 30% of the genomes. Additionally, genomes submitted to NCBI from 2018 onward contained a lower proportion of contaminant bases than those submitted prior to 2018. However, this trend has not continued: since 2018, the number of contaminated submissions has not declined and continues to rise in step with total submissions (Fig. 2c).

### Sources of contamination

**Fig. 3:**
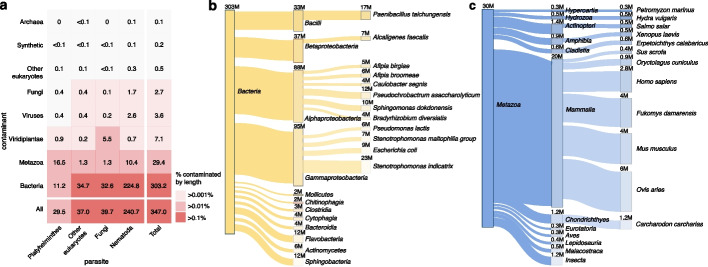
Sources of contamination in published parasite reference genomes identified using FCS-GX [19]. **a** Heatmap showing the total contamination (Mb) from different sources; grid colour indicates the proportion of each genome comprised of contamination. **b** Sankey plot of the top 12 bacterial contaminants at the species and order levels. **c** Sankey plot of the top 12 metazoan contaminants at the species and order levels. Numbers above taxonomic nodes indicate the total contaminant length (bp)

The vast majority (86%) of the contaminant sequences are of bacterial origin (Fig. 3a). Among the most abundant bacterial contaminants are nematode-associated species such as *Stenotrophomonas indicatrix*, and *Sphingomonas* spp., which were identified in nematode genomes (Fig. 3b). Both of these species are part of the CeMbio kit that can be purchased through the Caenorhabditis Genetics Center (CGC) to inoculate *Caenorhabditis elegans* with a simplified nematode microbiome [25]. Other nematode-associated contaminant species include *Alcaligenes* spp. and *Pseudochrobactrum* spp., which are known to form part of the *Steinernema* microbiome [26] and were found to be the top bacterial contaminants in the *Steinernema* genomes we screened. Interestingly, we also identified the nematicidal *Paenibacillus* spp. as a contaminant in *C. elegans* genomes [27]. In addition, we identified several bacterial species associated with the host of the parasite, such as the porcine pathogen *Chlamydia suis* in *Trichuris suis*, which was isolated from a pig (*Sus scrofa*), as well as common gut microbes like *Escherichia coli* and *Morganella morganii* in intestinal parasites such as *Trichuris trichiura*, *Anisakis simplex*, and *Baylisascaris schroederi*. Laboratory protocols were found to be another source of contamination with several contaminants, such as *Bradyrhizobium* spp., *Afipia* spp., and *Caulobacter* spp. having been detected in ultra-pure water and DNA kits used for DNA extraction [28, 29].

Metazoans are the second most abundant contaminant source, accounting for 8.4% (29.4 Mb) of the total contamination. For example, Platyhelminthes (flatworms) contained a total of 16.5 Mb of metazoan DNA (Fig. 3a). Most of this contamination can be traced back to the host from which the parasite specimen was taken, as was the case with the human filarial parasite *Mansonella sp. 'DEUX'* genome, which contained 653,059 bases of human DNA. Similarly, the two *Schistosoma japonicum* genomes contained 2,718,136 bases of house mouse (*Mus musculus*) and 600,660 bases of European rabbit (*Oryctolagus cuniculus*) DNA, respectively, while the *Taenia solium* genome contained 150,127 bases of pig (*Sus scrofa*) DNA (Fig. 3c). In all of these cases, the identified contaminant matched the host information that was provided in the genome metadata. In other words, the contaminant in these cases was likely the host from which the parasites were sampled.

Even when the identified vertebrate contaminant did not initially match the provided host information, further examination frequently revealed the host to be the actual source of contamination, as the contaminant was misidentified. For example, the Damara mole-rat (*Fukomys damarensis*), an African rodent, was found to be among the most abundant metazoan contaminants due to its presence in the genome of the flatworm *Echinococcus oligarthrus* extracted from an Azara’s agouti (*Dasyprocta azarae*) specimen in Argentina. However, closer inspection using a comprehensive rodent database (see “Methods”) revealed that more than 99% of the contaminant sequences matched the Central American agouti (*Dasyprocta punctata*), a close relative of the Azara’s agouti. Since no reference genome is available for this species, we suspect the putative contamination originated from the host. Similarly, the sheep (*Ovis aries*) contamination in the *Onchocerca flexuosa* and *Parelaphostrongylus tenuis* genomes proved to be red deer (*Cervus elaphus*) and white-tailed deer (*Odocoileus virginianus*), respectively, which are the host species from which the corresponding parasite specimens were sampled. No host information was provided for the bovine parasite *Onchocerca ochengi*, but we suspect that the sheep (*Ovis aries*) contamination we identified originated from cattle (*Bos taurus*) and was misclassified due to cross-mapping among closely related bovids (Additional File 1: Table S4).

### Screening results from simulated data

**Fig. 4:**
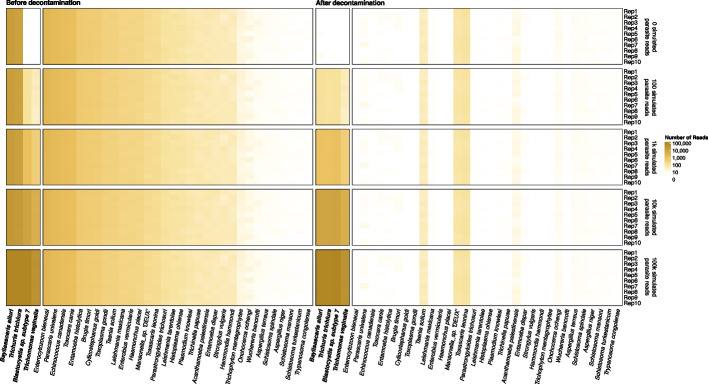
Reference decontamination preserves sensitivity while reducing contamination-driven false positives. Five coprolite-like datasets (10 replicates) were simulated with increasing spike-ins of four target parasites (in bold) (see “Methods”; Additional File 1: Table S5). Heatmaps show read assignments before (left) and after (right) decontamination (color intensity = assigned reads). True-positive signal for targets (in bold) is retained, while false positives are reduced substantially (~ 30–100 ×) after decontamination

To assess the sensitivity and specificity of our method, we simulated five coprolite datasets with varying levels of parasite DNA and a constant background of microbial, human, and pig DNA. To evaluate performance across different contamination levels, we included two parasite species with substantial (2–4 Mb) levels of contamination (*Baylisascaris ailuri* and *Trichuris trichiura*) and two species with minimal (< 50 kb) contamination (*Blastocystis subtype 6* and *Trichomonas vaginalis*). Using the original, unfiltered reference database, we screened the data with a k-mer-based classification pipeline (see “Methods”) and observed a high number of contaminant-related misalignments, averaging around 38,000 per replicate (Fig. 4, Additional File 1: Table S6). This was reduced to around 200 contaminant-related misalignments per replicate using the decontaminated version of the database (Fig. 4, Additional File 1: Table S7). Most of these sequences were aligned to *Taenia solium*, *Toxascaris leonina*, and *Mansonella sp. ‘DEUX’*, where the source of the misaligned reads matched either the host species (e.g., pig/human) or the host gut microbiome, indicating that not all contaminant sequences could be removed (see Additional File 1: Table S8).

In terms of our ability to detect true positives, we observed that for the simulated species *B. ailuri*, *T. trichiura*, and *Blastocystis subtype 6*, over 95% of reads were correctly assigned before and after filtering, demonstrating that the decontaminated database maintains high sensitivity. An exception was *T. vaginalis*, which showed reduced assignment efficiency—only ~ 40% of simulated reads were retained—likely due to its exceptionally high repeat content (> 65%) [30]. On the other hand, decontamination resulted in substantially fewer contamination-related misalignments in the simulated parasite genomes (Additional File 1: Table S9). Overall, this translates into a substantial improvement in precision, increasing from 7.9% to 94.3% in replicates with 1,000 parasite reads per species (Additional File 1: Table S9).

### Screening results from published datasets

To evaluate our approach on real-world data, we applied it to 14 previously published metagenomic datasets comprising both ancient and modern samples (Additional File 1: Table S10). The ancient datasets include material from a range of substrates, such as latrine sediments [31], human and dog coprolites [32–38], and human mummified remains from the Tyrolean Iceman [39, 40] and an 18th-century individual [41]. The modern datasets consist of stool samples from Hadza hunter-gatherers in Tanzania [42], two rural communities in northeastern Madagascar [43], and pigs from European slaughter farms [44]. We screened these datasets using both the original parasite genome reference database and our decontaminated version, in which contaminant sequences were removed (see “Methods”). To distinguish true positives from false positives, we assessed coverage evenness and entropy, with uneven distributions interpreted as signs of contamination and even coverage supporting genuine detection [45, 46]. Our simulations showed that alignments with > 50% contaminant reads consistently displayed uneven coverage entropy scores, and that in most cases, 1,000 mapped reads were sufficient to evaluate these patterns (Additional File 1: Table S9; Additional File 2: Fig. S3).

**Fig. 5:**
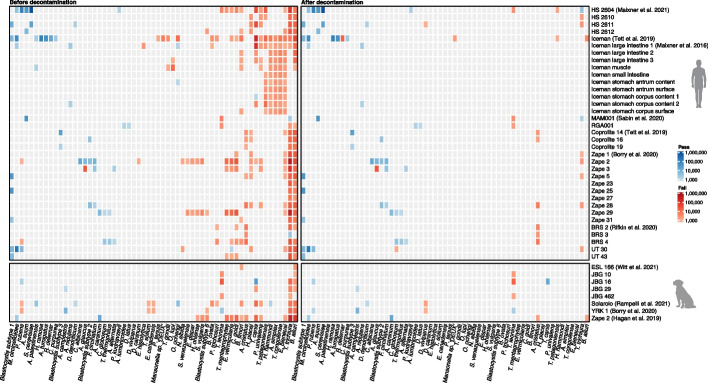
Decontamination of reference genomes leads to improved precision and overall accuracy of parasite detections. Using real data, we show that decontaminating reference genomes dramatically reduces the number of contamination-related misalignments with uneven coverage (red/fail) while the number of likely true positive detections with even coverage (blue/pass) remains unchanged, demonstrating that decontamination enhances the specificity of parasite detection without compromising sensitivity

In the ancient datasets, using the original reference database, we identified 322 putative parasite hits across 44 samples, corresponding to 59 species (Additional File 1: Table S11). However, 255 of these (79%) failed the evenness of coverage criterion, amounting to 3.1 million false positive alignments out of 5.1 million total. The majority (1.6 million reads) were assigned to *Trichuris trichiura*, including three samples (HS2604, HS2611, and HS2612) from the Hallstatt salt mines [34]. These were flagged as false positives due to their highly uneven coverage, despite declining edit distance distributions (Additional File 2: Fig. S4). When reanalysed using the decontaminated database, the number of uneven alignments decreased by 97%, from 3.1 million to 0.1 million, while the number of evenly distributed alignments remained stable at approximately 1.9 million (Additional File 1: Table S12). Notably, for sample HS2604, decontamination converted the previously uneven *T. trichiura* alignment into one with even coverage—consistent with additional support from Maixner et al. [34], who recovered a *T. trichiura* mitochondrial genome and reported hits to the cytochrome c oxidase subunit I gene for this sample. A BLAST search of the assigned *T. trichiura* sequences suggested that the original uneven alignment was due to contamination with *Shigella *spp*./E. coli* (Additional File 1: Table S13). In addition, we identified other likely true positives, including *Ascaris suum* and *Ascaris lumbricoides* in latrine samples from medieval Riga and Jerusalem [31], the lancet liver fluke, *Dicrocoelium dendriticum,* in one of the Hallstatt coprolites [34], and the canine parasites *Ancylostoma caninum*, *Toxocara canis*, and *Toxascaris leonina* in dog coprolites from Mexico [37], Yorkshire [32], and Illinois [38], respectively (Fig. 5).

In the modern datasets, a similar pattern emerged. In the Hadza dataset [42], 22.6 million reads aligned to genomes in the original database with highly uneven coverage; in contrast with ParaRef, this dropped to 0.3 million. Of the 22.6 million original alignments, 16.7 million had been falsely attributed to *T. trichiura*. Conversely, with ParaRef we recovered previously undetected, likely true positives: 16 cases of the blood fluke *Schistosoma mansoni*, two cases each of *Taenia saginata* and *Taenia solium*, and one case of *Enterobius vermicularis*, a parasite commonly found in children [47] (Additional File 2: Fig. S5).

In the Madagascar samples [43], we obtained 544 parasite hits (totalling 16 million reads with uneven coverage) using the original database, which was reduced to 57 hits (0.3 million reads) with ParaRef. Confirmed positives included 30 cases of *Blastocystis *spp*.* and one case of *Ancylostoma ceylanicum*, a zoonotic hookworm prevalent in Madagascar and Southeast Asia [48, 49] (Additional File 2: Fig. S6). Lastly, in the pig farm dataset [44], uneven alignments dropped from 16.6 million to 0.2 million reads after decontamination. Alarmingly, 123,000 of the original alignments could be traced back to the host (pig) contamination present in the *T. solium* genome despite the removal of host DNA from the dataset before assignment, indicating that filtering out host DNA alone is insufficient. With ParaRef, *T. solium* reads were no longer detected; instead, we identified three cases of *Ascaris suum* and one of *Oesophagostomum dentatum* [50] (Additional file 2: Fig. S4).

## Discussion

We identified widespread contamination in publicly available parasite reference genomes, which can lead to inflated false-positive detections in metagenomic screens—especially in complex matrices (coprolites, latrine sediments, faecal material)—and can evade standard validation (edit-distance distributions, ancient DNA damage). To address this, we curated ParaRef, a decontaminated parasite reference database. Applied to simulated and published datasets, ParaRef markedly reduced contamination-driven misassignments while maintaining sensitivity. These improvements are consequential for both ancient DNA research and modern clinical and veterinary metagenomics, where misidentifications can mislead inference and diagnosis.

Of the 831 parasite genomes assessed, 262 (32%) each contained > 100 kb of contaminant sequence. Strikingly, several of the most affected references are major human pathogens—*Schistosoma* spp. and *Trypanosoma cruzi*—with direct implications for diagnosis, treatment, and epidemiological surveillance [51]. Even the primary malaria agents (*Plasmodium falciparum*, *P. vivax*, *P. malariae*) carry detectable contamination, underscoring how pervasive—and hard to recognize—this problem is. As metagenomics is increasingly deployed to detect low-titer infections, asymptomatic carriage, and environmental reservoirs, even low-level contamination becomes a consequential confounder [52, 53].

Despite advances in long-read sequencing and improved assembly algorithms [54, 55], the rate of contamination in public reference genomes does not appear to be declining. Assemblies at the contig or scaffold level remain the most affected and are often the only available resources for many parasite species. As reported previously [19], we found that short contigs—particularly those under 1 kb—harboured disproportionately high levels of contamination, capturing ~ 15% of all detected contaminant bases while representing only < 2% of total assembly length.

To mitigate these issues, we decontaminated references with FCS-GX [19] and Conterminator [23], removed flagged regions, and excluded contigs < 1 kb prior to classification. Applying these curated references to simulated and published datasets reduced contamination-driven assignments by 30–100 × relative to the unfiltered NCBI set in our setup (KrakenUniq with alignment-based validation). Importantly, this noise reduction did not come at the cost of sensitivity: true-positive read counts remained stable across conditions (Fig. 5). Although some horizontally transferred genes may be flagged and removed, species-level calls were effectively unchanged. For end-users of ParaRef, we recommend retaining downstream validation: confirm taxonomic identity via average nucleotide identity (ANI), alignment metrics (e.g., mode edit distance), genome-wide evenness/entropy of coverage, and—when analysing ancient DNA—assess deamination and fragment-length profiles.

While we did not set out to systematically benchmark decontamination tools, using both FCS-GX [19] and Conterminator [23] yielded consistent observations worth noting. Conterminator flagged contamination in more genomes—likely reflecting its broad microbial reference set and its practice of marking cross-kingdom sequence sharing (including potential horizontal gene transfer)—and it supports custom databases; however, it does not infer contamination sources and, in our setup, required iterative runs with manual removal (see “Methods”). FCS-GX, by contrast, typically identified more contaminant bases per genome, automated both detection and filtering, and reported the most likely taxonomic origin of the contaminant, but it relies on a fixed NCBI reference offering less flexibility. In practice, FCS-GX was the most convenient for rapid decontamination, whereas Conterminator provided complementary sensitivity when a tailored search space was needed; using both in combination gave the most comprehensive curation.

Turning to the origins of contamination, the dominant contributors are gut microbes and host DNA—both abundant in the sample types most often screened for parasites (faeces, host tissue). This overlap increases the risk that contaminant reads are misinterpreted as true positives. For example, gut-microbial contamination in the *Trichuris trichiura* and *Baylisascaris schroederi* reference genomes produced frequent false positives in our faecal datasets, which contain high levels of *Escherichia coli* and other common gut bacteria. Host DNA contamination poses a similar challenge, even when parasite specimens are carefully isolated. Pig cells have been observed inside the spiral canal of *Taenia solium cysticerci* [56], and we detected > 150 kb of pig DNA in the *T. solium* reference, yielding dozens of false positives in the pig dataset despite host-read removal during preprocessing.

Ancient DNA studies face additional complications. When contaminant DNA is itself ancient—such as human or microbial DNA preserved in coprolites—it can display characteristic ancient DNA signatures, including cytosine deamination and short fragment lengths. These features are typically used to authenticate ancient sequences and may therefore give false confidence in spurious parasite detections. In such cases, contamination-related misalignments can only be reliably identified by assessing the coverage evenness and entropy of the aligned reads. Interestingly, reference genome contamination can also lead to false negatives: if the parasite is genuinely present but the genome contains contaminated regions, reads may be unevenly distributed, suggesting a false positive hit. This was the case for *T. trichiura* in the Hallstatt coprolite sample HS2604 [34], which initially failed coverage-based validation but was determined to be a likely true positive hit following reference genome decontamination.

In addition to improving detection accuracy, decontamination revealed previously unreported taxa in ancient datasets. We detected the ruminant parasite *Dicrocoelium dendriticum* in a Hallstatt coprolite (HS2611) [34], likely reflecting ingestion of infected beef, and the human whipworm *Trichuris trichiura* in gut tissue from an 18th-century individual from Basel [41]. Although *T. trichiura* has been reported in the Tyrolean Iceman by microscopy [57], we did not recover its DNA from the published metagenomic data [39, 40]. Instead, we identified two fungal *Aspergillus* spp., consistent with Oskolkov et al. [58], as well as two previously unreported opportunistic pathogens, *Lichtheimia ramosa* and *Blastocystis* [59, 60]. We also detected canine parasites—*Ancylostoma caninum*, *Toxocara canis*, *Toxascaris leonina*—in dog coprolites from Yorkshire [32], Mexico [37], and Illinois [38], underscoring the value of ancient metagenomics for tracing parasite transmission and host co-evolution.

Complementing these findings, our reanalysis of modern metagenomic datasets also revealed several previously undetected parasite infections in clinical and veterinary settings. For example, screening pig stool samples [61] confirmed the presence of the large pig roundworm *Ascaris suum* as well as an unreported case of *Oesophagostomum dentatum*, while the analysis of stool samples from Hadza hunter-gatherers [42], revealed multiple instances of *Schistosoma mansoni* infection—a major cause of schistosomiasis in Africa [62]—as well as several cases of *Taenia* spp. infection and one case of *Enterobius vermicularis*. In addition, the dataset from northeastern Madagascar [43] revealed several instances of *Blastocystis* spp. infection and a previously undetected case of hookworm (*Ancylostoma ceylanicum*) infection, common in Southeast Asia and Africa [48]. These results illustrate the utility of metagenomic parasite screening in both clinical and ecological studies, where reliable species-level identification can inform diagnostics, patient care, and epidemiological surveillance.

Taken together, our results show that reference decontamination markedly improves specificity while preserving sensitivity across ancient and modern datasets. Although we used a specific workflow [63], the issues we highlight apply to all reference-based classifiers: in k-mer methods, contaminated regions generate spurious k-mers that misassign reads, while in alignment-based workflows, contaminated sequence can produce high-scoring hits that are hard to distinguish from genuine matches unless genome-wide read distribution is evaluated. Importantly, because k-mer classifiers assign reads based on short sequence matches alone, an additional alignment step is needed to validate assignments and detect potential contamination [45].

One strategy to mitigate these issues is to use more targeted databases, such as those based on marker genes or protein sequences. While this reduces the risk of contamination, it also limits taxonomic resolution and sensitivity, making it difficult to detect low-abundance DNA or assess ancient DNA damage patterns. Alternatively, comprehensive whole-genome databases that include reference genomes from all kingdoms can help reduce misalignments by allowing contaminant reads to align to their true source and be assigned to the lowest common ancestor (LCA) node. However, these large databases are computationally demanding and may not include reference genomes of all potential contaminant sources.

Ultimately, no method is entirely immune to the effects of reference contamination. Even with curated databases, cross-kingdom sequence homology, low-complexity regions, and taxonomic misassignments remain significant obstacles [64, 65]. Therefore, we recommend a tiered strategy: use of decontaminated whole-genome references wherever possible, combined with post-assignment filters such as coverage evenness scores, and additional validation for high-confidence hits. In practice, we advise requiring ≥ 1,000 aligned reads per candidate detection to enable robust calculation of evenness/entropy metrics, edit-distance distributions, and—where applicable—ancient DNA deamination and fragment-length profiles. Comprehensive databases that include references from all kingdoms can further reduce erroneous assignments by providing appropriate targets for reads that might otherwise align spuriously to contaminated sequences. In addition, we recommend excluding contigs < 1 kb, as these were disproportionately affected by contamination in our dataset. This approach will result in more robust parasite detection across a broad range of metagenomic applications.

## Conclusions

Our study highlights the pervasive issue of contamination in public databases and its detrimental impact on the accuracy of parasite detection in metagenomic studies. By removing contaminant sequences from 831 parasite genomes, we built ParaRef, which substantially reduces contamination-driven false positives without sacrificing sensitivity across simulated and published datasets. Because the error originates at the reference level, these gains are independent of classifier choice—applying to both k-mer and alignment-based workflows. We recommend pairing ParaRef with routine post-assignment checks—coverage evenness/entropy, edit-distance profiles, and (for ancient DNA) deamination and fragment-length patterns—to ensure robust species-level calls. ParaRef enables more accurate detection in clinical and veterinary practice and strengthens archaeoparasitological inference, advancing our understanding of both contemporary and historical parasitic infections.

## Methods

### Compilation of reference genomes

A total of 831 genomes were compiled on January 24 2023 (Additional File 1: Table S1), consisting of all available *Platyhelminthes* (n = 100), *Nematoda* (n = 338), and *Blastocystis* (n = 9) genomes from NCBI as well all *Fungi* (n = 227) and eukaryotic pathogen (n = 157) genomes from VEUPathDB Release 61 [66]. The metadata for the downloaded genomes was accessed using the command-line tool NCBI DATASETS [67]. Some of the included species are free-living or infect plants or insects and are thus of lesser importance for archaeoparasitological studies, but were included as decoy species to decrease the risk of false positive environmental hits.

### Decontamination of reference genomes

Adapter and vector sequences were removed from the reference genomes using FCS-adaptor [68]. FCS-GX [19] was then used on default settings on the resulting fasta files to identify and remove regions in the parasite genomes that are of exogenous origin. In order to confirm the results of FCS-GX [19] and to remove potentially undetected contamination, Conterminator [23] was employed on the raw reference genomes. In order to compile the reference dataset for Conterminator [23], all representative and/or complete bacterial and archaeal reference genomes were downloaded from RefSeq in January 2023 in addition to the hg38 (GCF_000001405.26) and T2T (GCF_009914755.1) human reference genomes and five animal host species (dog (GCF_000002285.5), cat (GCF_018350175.1), sheep (GCF_016772045.1), cattle (GCF_002263795.2), pig (GCF_000003025.6)). Two different human genomes were used, as the recently published T2T genome is a more comprehensive representation than the hg38 genome, which contains alternate haplotypes present. A non-metazoan taxonomic ID was assigned to the metazoan sequences to allow the detection of metazoan host contamination in metazoan parasite genomes. Conterminator [23] was adapted to identify contaminating regions that stretch longer than 20,000 bases. Contigs and scaffolds that are composed of more than 50% contamination were removed, and the remaining identified regions were hard-masked using bedtools maskfasta [69]. The detection of contamination using Conterminator [23] and subsequent removal and masking of regions was done iteratively until no contaminant sequences could be detected.

### Screening of simulated datasets

To assess the effect of reference contamination on the detection of parasites in metagenomic studies and to evaluate the effectiveness of the decontamination procedure, we simulated coprolite shotgun data that contained different abundances of four parasites. We compiled a parasite database consisting of one genome per species, using either the representative genome or, in cases where no representative genome was available, the genome with the highest assembly level and lowest contamination estimate. To evaluate the efficacy of the decontamination, we masked low-complexity regions and built separate KrakenUniq databases for the masked original and decontaminated genomes. We then used gargammel [70] to simulate 100 million sequences of background coprolite data, consisting mostly of bacteria and archaea identified in HS2604 by Maixner et al. [34], 2% human DNA (GCF_009914755.1), and 1% pig DNA (GCF_000003025.6), using the length distribution from the HS2604 sample. We then added 0, 100, 1,000, 10,000 and 100,000 sequences from the decontaminated genomes of *B. ailuri*, *Blastocystis subtype 7*, *T. trichiura* and *T. vaginalis*, respectively. Each of the five datasets was run in 10 replicates and screened with the Snakemake-based *pathopipe* workflow [46], which uses KrakenUniq [63] for metagenomic classification, followed by genus-level read mapping with Bowtie2 [71], and summary statistics for validation of pathogen hits retaining hits with over 300 unique kmers. For each hit, reads were extracted at genus level and aligned to all reference genomes of the respective genus using Bowtie2 v2.5.3 [71] with “global very sensitive” mode enabled and allowing 1 mismatch for the seed alignment. The alignments were deduplicated using samtools markup [72] and mapping statistics such as average mapping quality and breadth of coverage were computed for each alignment with R [73]. Precision was calculated by dividing the number of aligned simulated target reads by the number of all aligned per species, as well as per replicate.

### Screening of ancient and modern metagenomic datasets

The datasets were downloaded from ENA using accession codes from AncientMetagenomeDir [74]. The data were preprocessed and host DNA removed by aligning to the respective host organism using the nf-core/eager 2.4.7 pipeline [75]. Taxonomic classification as described above, using both the raw and decontaminated database. From each genus, only the species with the highest number of unique kmers was selected. We validated hits with more than 1,000 aligned reads from genera that include parasitic species which infect humans or livestock, using edit distance (mode edit distance less than 2), ancient DNA damage patterns [76], and evenness of coverage scores. For the evenness of coverage, the coverage entropy score (covPosRelEntropy1000) was used, which transforms the frequency of sequences in a 1,000 bp window into Shannon entropy values, which are then normalized against the maximum possible entropy. Genomes with a covPosRelEntropy1000 above 0.8 were then defined as being evenly distributed. We used the number of genomes with more than 1,000 aligned sequences as well as differences in read assignments and coverage statistics as metrics to assess the efficiency of the contamination masking.

### Taxonomic reclassification using BLAST

To resolve the discrepancy between the metazoan species identification of contamination detected with FCS-GX [19] and the specimens’ host organism, we extracted all contigs from GCA_900683695.1 that were marked as coming from the *Fukomys damarensis* genome, and contigs with *Ovis aries* contamination from genomes that were predicted to have more than 100,000 bases from the *Ovis aries* genome, i.e. GCA_002249935.1, GCA_019055375.1, and GCA_000950515.2. We built a blastn database for all Rodentia and Pecora representative genomes respectively and performed a nucleotide-to-nucleotide search between the contaminated contigs and the Rodentia/Pecora databases using megablast [77]. For each contig, we report the best fitting match, i.e. the match with the lowest e-value (Additional File 1: Table S4). We used the same approach to determine the source of the contaminant reads in the *T. trichiura* alignment file for one of the Hallstatt coprolites (HS2604) [34]. We downloaded the core_nt database from BLAST (August 2024) and extracted the quality-filtered and deduplicated sequences from the *T. trichiura* non-decontaminated alignment file of HS2604. We then performed a nucleotide-to-nucleotide megablast [77] search between the extracted sequences and the core_nt database as described above to find the best-fitting match for each sequence (Additional File 1: Table S13).

## Supplementary Information

Additional file 1: Supplementary Tables S1-S13. Summary statistics of taxonomic classifications and additional information to the samples, reference genomes, and datasets used in this study.

Additional file 2: Supplementary Figures Fig S1-S7. Additional figures not included in the main text.

## Data Availability

All data and code for this study are available on Github at https://github.com/schroeder-group/ParaRef [78]. ParaRef, the decontaminated parasites database generated in this study, can be downloaded from Zenodo (10.5281/zenodo.13744644) [79].
