# Supplementary material for: ParaRef: a decontaminated reference database for parasite detection in ancient and modern metagenomic datasets
Source: Genome Biol. 2025 Oct 23;26:365. doi: 10.1186/s13059-025-03818-w (PMC12548146; doi:10.1186/s13059-025-03818-w)
Supplement: Supplementary file 2 — Additional file 2: Supplementary Figures Fig S1-S7. Additional figures not included in the main text. [file 13059_2025_3818_MOESM2_ESM.pdf]

Additional File 2: Supplementary Figures

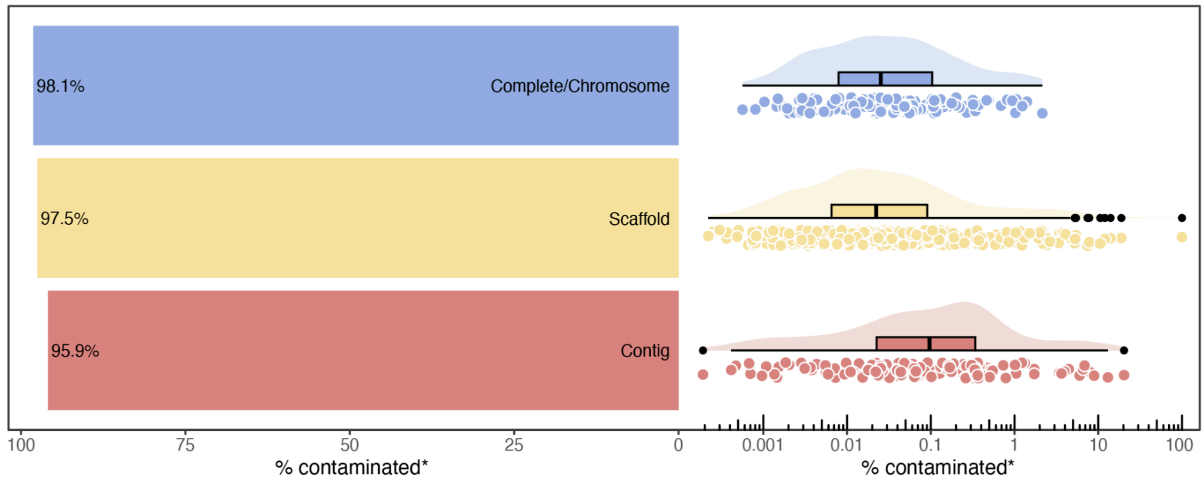

Fig. S1. Extent of contamination in published parasite genomes detected with Conterminator [23]. Proportion of published parasite genomes flagged as contaminated by assembly level (left); Fraction of contaminant bases in each parasite genome (right). Each genome is plotted individually as a circle. *As discussed in the main text, Conterminator [23] likely overestimates the amount of contamination due to the way it identifies contaminant sequences.

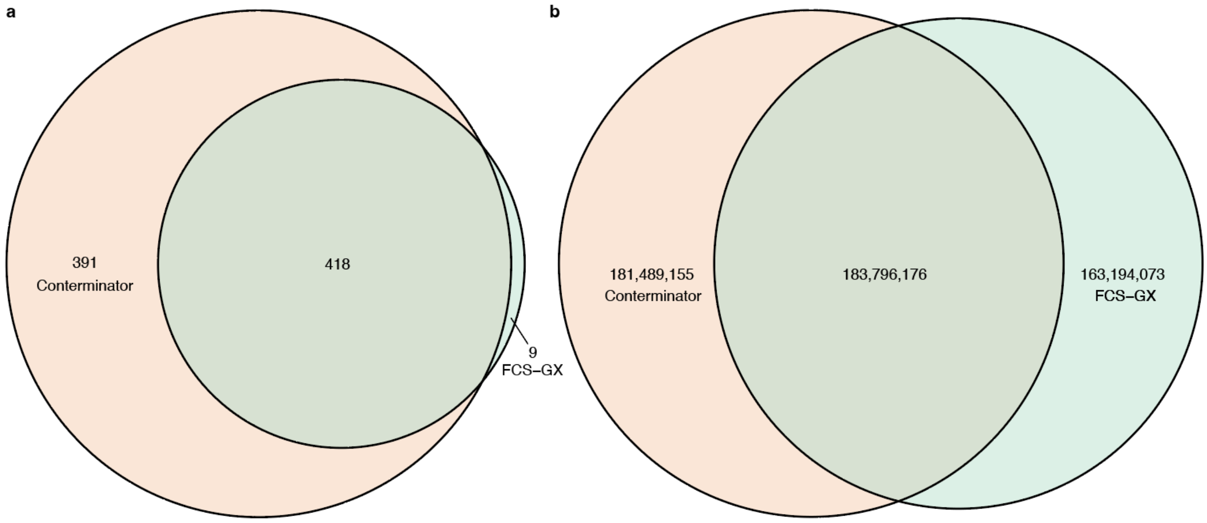

Fig. S2. Conterminator vs. FCS-GX. a) Number of published reference genomes flagged as contaminated by Conterminator [23] and FCS-GX [19]; **b)** Number of bases identified as contamination by Conterminator [23] and FCS-GX [19].

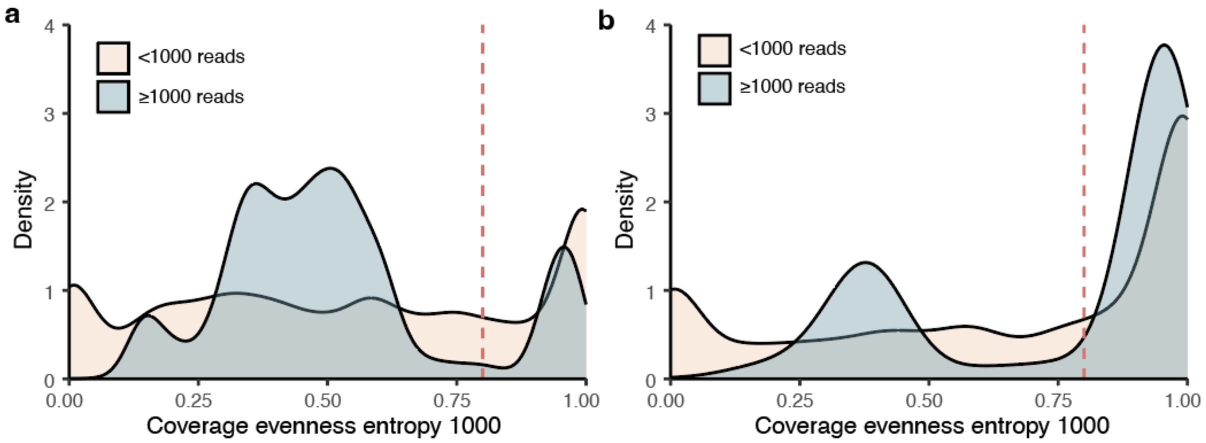

Fig. S3. Distribution of evenness of coverage entropy scores (covPosRelEntropy1000) for identified parasite genomes in ancient and modern datasets with less than 1,000 assigned reads (beige) and more than 1,000 aligned reads (blue) before **(a)** and after **(b)** decontamination.

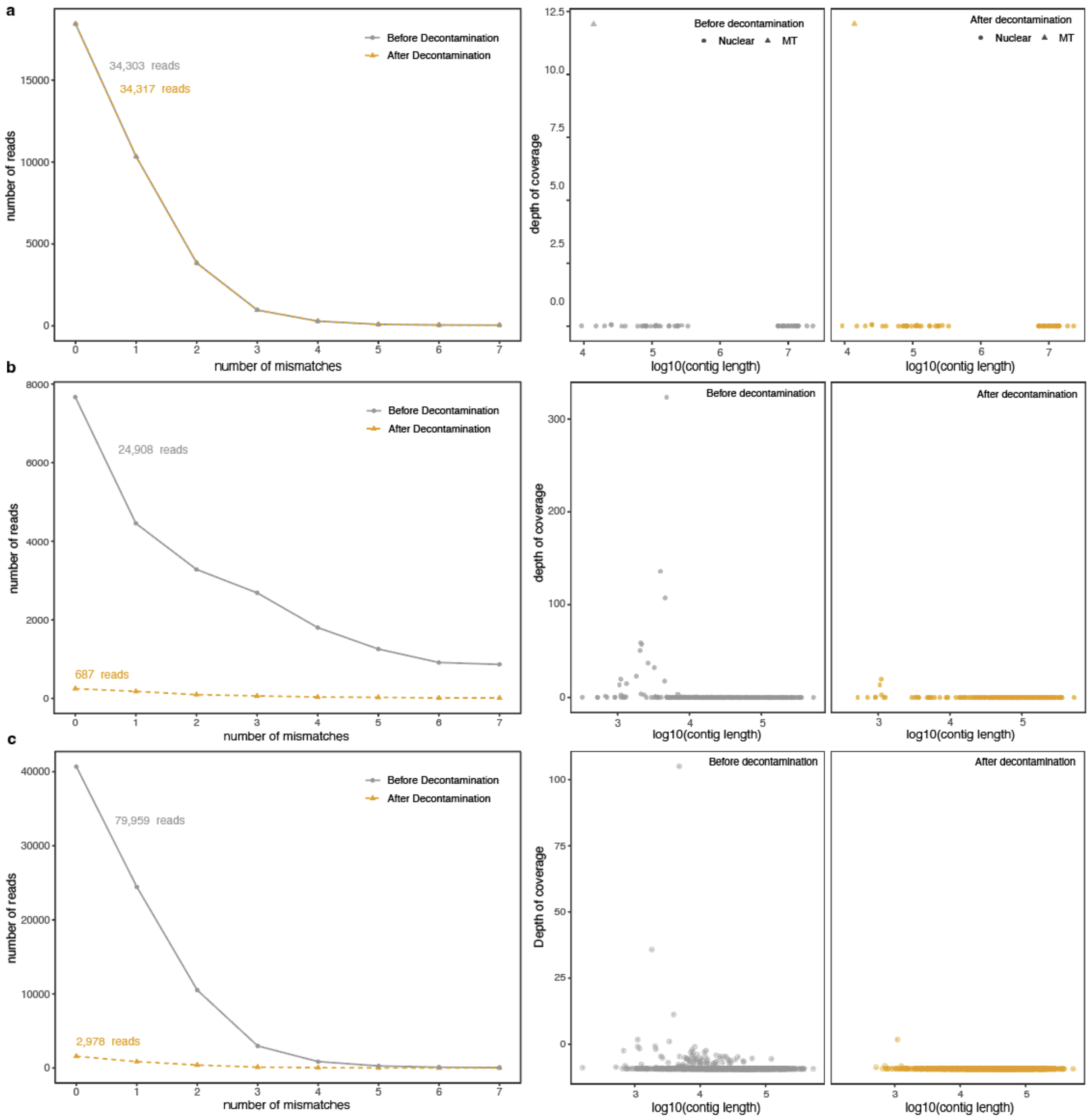

Fig. S4. Change in edit distance distribution (left) and depth of coverage across all contigs (right) before and after decontamination in three parasite hits detected in coprolite samples from Hallstatt [34]. a) *Ascaris suum* detected in sample HS2604, displaying a declining edit distance distribution and little variation in nuclear depth of coverage, suggesting that *A. suum* is a true positive. **b)** *Trichuris trichiura* detected in coprolite HS2612. While displaying a declining edit distance distribution like potential true positives, reads are unevenly spread across contigs - a hallmark of contamination. **c)** *T. trichiura* detected in HS2604 with both parasite and contaminant DNA, and therefore failing validation criteria before decontamination, and being a potential true positive after decontamination.

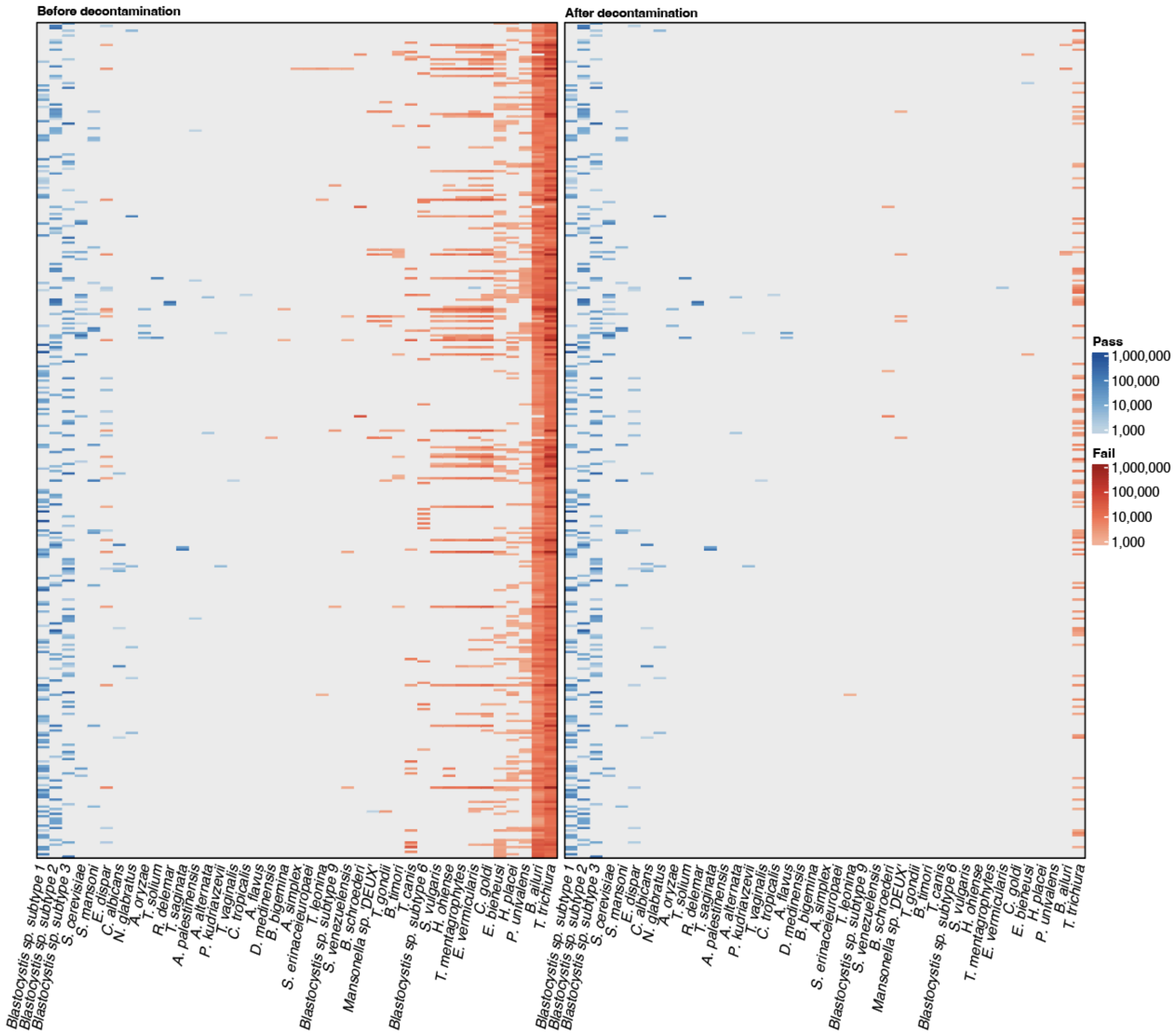

Fig. S5. Heatmap showing parasite detections in deeply sequenced gut metagenomes of Hadza hunter-gatherers [42] before (left) and after decontamination (right). The color indicates whether the alignment had an even coverage (Pass) or not (Fail), with the intensity indicating the number of aligned sequences.

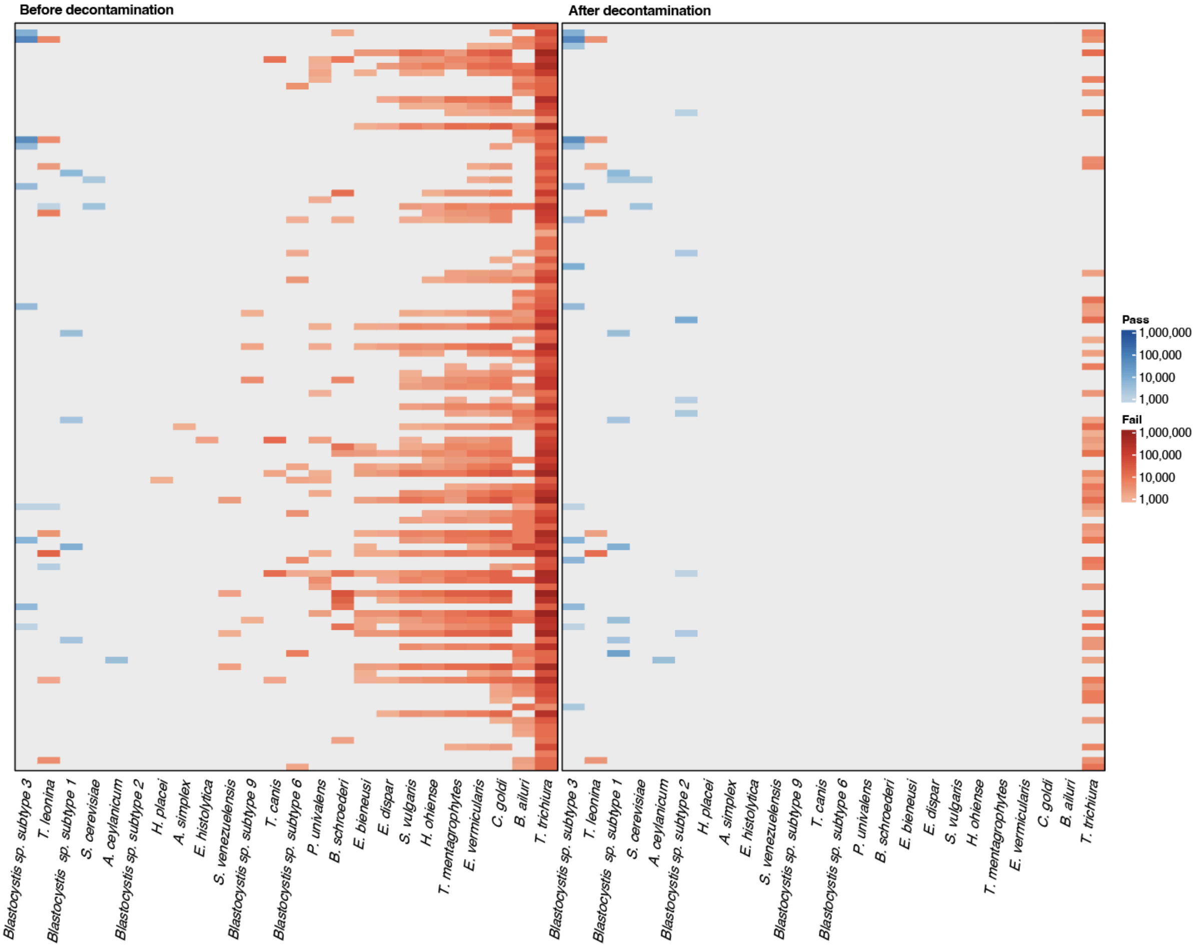

Fig. S6. Heatmap showing parasite detections in published Malagasy metagenomes [43] before (left) and after decontamination (right). The color indicates whether the alignment had an even coverage (Pass) or not (Fail), with the intensity indicating the number of aligned sequences.

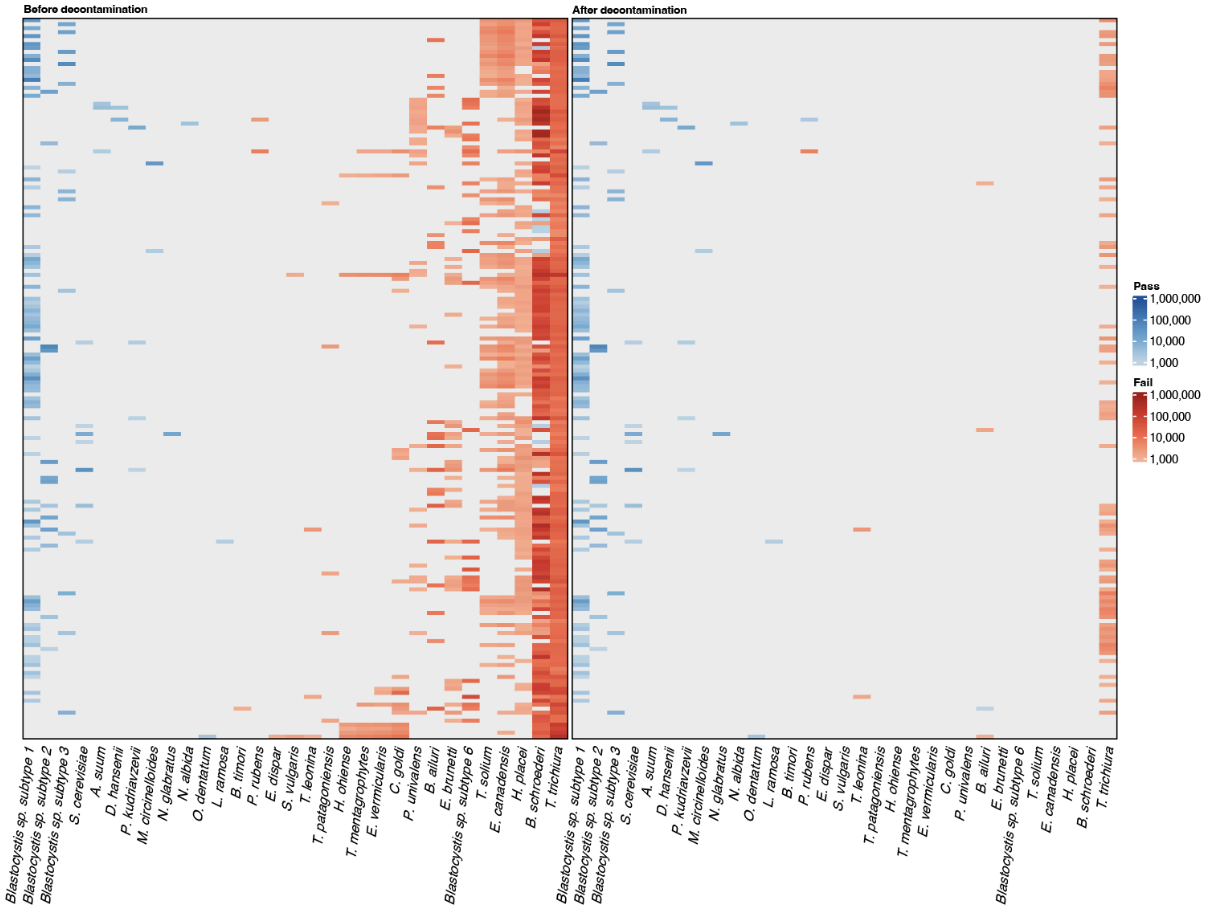

Fig. S7. Heatmap showing parasite detections in published metagenomic data from slaughter pigs [44] before (left) and after decontamination (right). The color indicates whether the alignment had an even coverage (Pass) or not (Fail), with the intensity indicating the number of aligned sequences.
